# Is Less More? The Need of Perioperative Antibiotic Prophylaxis and Burying K-Wires in Pediatric Trauma Surgery—A Retrospective Analysis of 779 Distal Forearm and Distal Humeral Osteosyntheses

**DOI:** 10.3390/children13081033

**Published:** 2026-08-03

**Authors:** Nadja Schmidt, Clemens Memmel, Christian Wulbrand, Alexander Hanke

**Affiliations:** 1 Department of Pediatric Surgery and Orthopedics, Clinic St. Hedwig, Barmherzige Brueder Regensburg, KUNO Pediatric University Medical Center, Steinmetzstraße 1-3, 93049 Regensburg, Germany; 2 Department of Trauma Surgery, University Medical Centre Regensburg, 93053 Regensburg, Germany; 3 Department for Trauma, Orthopedics and Sports Medicine, Hospital Barmherzige Brueder Regensburg, Prüfeninger Straße 86, 93049 Regensburg, Germany

**Keywords:** pediatric fracture, distal humerus, distal forearm, K-wire, perioperative antibiotic prophylaxis, PAP, surgical site infection, SSI, fracture-related infection, FRI

## Abstract

**Background:** K-wire osteosynthesis is a widely used, cost-effective technique for stabilizing pediatric and adolescent fractures. The beneficial effect of perioperative antibiotic prophylaxis (PAP) and burying the K-wires below skin level to reduce infectious complications is still controversial. The goal of this study was to evaluate if the omission of PAP and leaving K-wires unburied affected the complication rate. **Materials and Methods:** A retrospective analysis of pediatric and adolescent K-wire osteosyntheses over a period of 11 years was performed. Records were screened for general, infectious and implant-associated complications. Follow-up was performed until the implant was removed. **Results:** Between January 2014 and December 2024 a total of 1022 K-wire osteosyntheses were performed and 779 were eligible for this study. Median follow-up to implant removal was 30 days [28–36 days]. At least one complication was documented in 17.6% (137/779) of the cases. Infectious complications were low at 2.6%—including 11 (1.4%) wound healing disorders and 9 (1.2%) pin site infections—and did not show difference regardless of application of PAP. No cases of osteomyelitis were observed until follow-up. Furthermore, implant-associated complications were more frequent with buried wires (10.2%; 19/187) than with unburied wires (3.4%, 20/592). **Conclusions:** Omitting perioperative antibiotic prophylaxis and leaving K-wires unburied in pediatric and adolescent patients undergoing closed reduction and K-wire fixation of distal radial and distal humeral fractures does not seem to increase the risk for infectious complications. Furthermore, unburied K-wires can be removed without the need for a second general anesthesia. **Level of Evidence:** III (retrospective cohort study).

## 1. Introduction

At the beginning of the 20th century Martin Kirschner introduced the technique of using pointed wire osteosyntheses (K-wire) to treat fractures [1]. Due to its ease of use, short operating time and cost-effectiveness, closed reduction and K-wire osteosynthesis in combination with a cast is the leading procedure for stabilizing epiphyseal and metaphyseal fractures [2].

**Figure 1:**
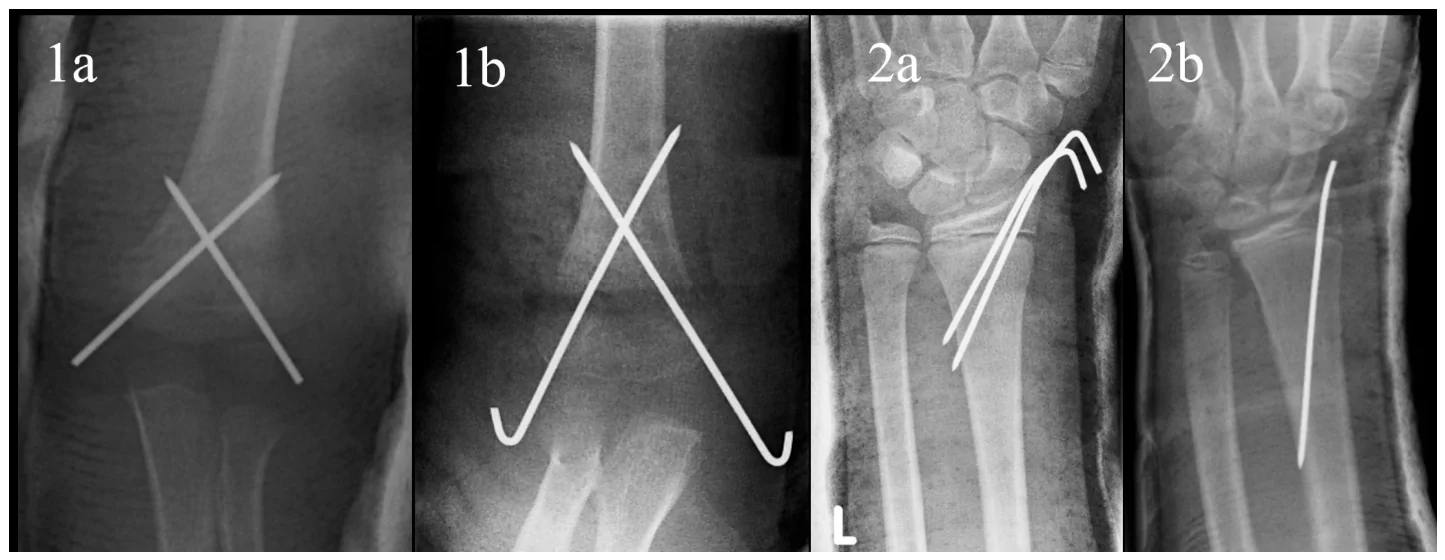
Radiographic examples of K-wire osteosyntheses: Buried K-wire osteosyntheses of the distal humerus (**1a**) and distal radius (**2a**) and unburied K-wire osteosyntheses of the distal humerus (**1b**) and distal radius (**2b**).

K-wires can be trimmed below the skin level (buried) and removed under general anesthesia. Alternatively, they can be left epicutaneously (unburied) and removed in an outpatient clinic without anesthesia or with minimal sedation (e.g., oral midazolam or inhaled nitrous oxide) (Figure 1). This eliminates the need for a second general anesthesia. Both methods have a similarly low complication rate and similar levels of parental satisfaction, particularly for fractures in the upper extremities [3,4,5,6,7,8]. Despite the benefits of this method, many wires are buried due to concerns about an increased infection rate [9].

A well-established method to prevent surgical site infections (SSIs) is the use of perioperative antibiotic prophylaxis (PAP). Currently, there are no guidelines for children, so clinical practice is often based on the guidelines for adults [10]. The current German guideline for adults recommends PAP for all closed fracture osteosyntheses, preferably a first- or second-generation cephalosporin [11]. A similar recommendation for PAP is also found in the American guideline on antimicrobial prophylaxis in surgery [12]. However, newer publications question the necessity for PAP in clean, minimally invasive surgery even with implants [13].

The infection rates reported in the literature following pediatric K-wire osteosyntheses vary considerably, ranging from 1.9% to 34.0% [14]. However, a recent study found no significant difference in the incidence of SSIs between patients with and without PAP after K-wire osteosyntheses of the distal humerus [15].

This retrospective analysis investigates whether the omission of PAP and leaving K-wires unburied affects the complication rate in pediatric osteosynthesis.

## 2. Materials and Methods

All fractures in children and adolescents (0–18 years) that were treated with K-wire osteosyntheses between January 2014 and December 2024 were included in this retrospective analysis. The data were collected at a single pediatric hospital affiliated with a Level I trauma center. Data were collected from the clinic’s IT system (SAP GUI v. 7.6.7, SAP Deutschland SE & Co. KG, Walldorf, Germany) and the digital radiological image archive (JiveX v. 5.4.0.4, Visus Health IT GmbH, Bochum, Germany). The primary variables of interest were the administration of PAP and the management of K-wire, whether they were buried or not. As unburied K-wire osteosynthesis was deemed clean and minimally invasive, the in-house standard was no PAP in case of closed reduction and K-wire osteosyntheses. If PAP was administered, it was given intravenously within 30 min before surgery. Until 2017, K-wires were buried below the skin and removed in general anesthesia. Following an institutional protocol change in 2017, K-wires were left unburied. Unburied wires were removed in the outpatient clinic without anesthesia or supported by inhalative nitrous-oxide sedation (Livopan 50/50%, Linde GmbH, 82049 Pullach, Germany).

Implant removal was used as follow-up. At this point, PAP-related complications should all be assessed and no additional information would be obtained by a longer follow-up. In particular, wound-healing disorders and late-onset osteomyelitis following implant removal performed at least 4 weeks after the initial surgery are unlikely to be influenced by the initial PAP application. The following parameters were obtained: demographics (gender, age and weight), fracture-specific information (date of accident, date of operation, AO fracture classification [16] and Gustillo Anderson classification [17] for open fractures), risk factors for fractures (e.g., refracture or secondary dislocation during a conservative treatment attempt), surgery-specific data (duration (min), type and dosage (mg) of PAP, buried or unburied K-wires, type of wound closure). As part of the follow-up the date of implant removal and the type of anesthesia used for the procedure (none, general anesthesia or nitrous oxide) were recorded. Complications were grouped as either infectious complications (e.g., wound healing disorders, pin site infections and osteomyelitis) or implant associated. The latter were further divided into complications associated with buried K-wires (e.g., perforation, irritation or implant failure) and those associated with unburied K-wires (e.g., ingrowth, irritation or implant failure). A wound healing disorder was defined as local abnormalities at the pin site (e.g., pressure ulcers or delayed wound healing), but without clinical signs of infection or the need for antibiotic treatment. Pin site infections were defined as the presence of inflammatory signs at the pin site (e.g., redness, swelling or purulent discharge), requiring oral antibiotic treatment. Osteomyelitis was defined as a deep bone infection, confirmed clinically and radiologically, requiring surgical treatment and/or prolonged systemic antibiotic treatment. Furthermore, general surgical complications (motor function deficits, sensory disturbances, nerve lesions/paresis, insufficient consolidation or consolidation in malposition) and cast-related problems were assessed.

**Figure 2:**
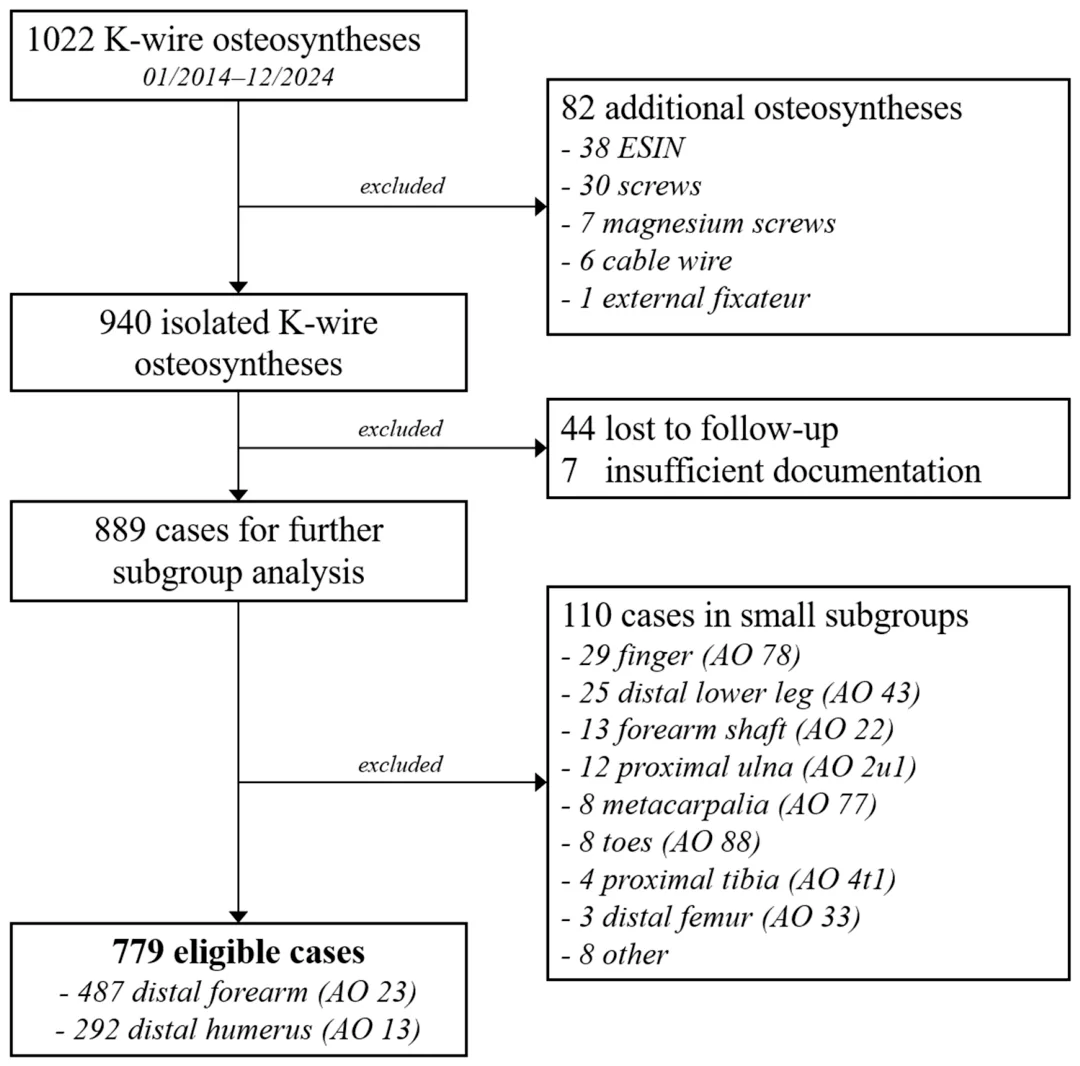
Flowchart of case selection and exclusion process for K-wire osteosyntheses included in this study. Due to statistical comparability, anatomical regions that would create subgroups with less than 10% of the total were excluded. ESIN = Elastic Stable Intramedullary Nailing.

Any datasets without follow-up or lacking documentation of any of the above information were excluded. The statistical analysis was limited to the two main anatomical sites: the distal forearm (AO 23) and the distal humerus (AO 13). The other fracture sites occurred in too few cases and were therefore excluded (Figure 2).

Data analysis was performed using statistical analysis software (SPSS v. 30.0, IBM SPSS Inc., Chicago, IL, USA). All data were tested for normal distribution using the Shapiro-Wilk test. Non-parametric results are presented as median [interquartile range]. Group comparisons were performed using the Mann–Whitney U test. Non-parametric data were analyzed using either the Chi-square test or Fisher’s exact test with Monte Carlo simulation. It is worth noting that no normal distribution of all the data was found, so no parametric tests were used. The overall significance level was set at *p* = 0.05.

## 3. Results

### 3.1. Study Population

A total of 1022 K-wire osteosyntheses were performed on 1005 patients (12 with the same accident and different fracture locations, 5 with different accidents). Of these, 8.0% (82/1022) were excluded due to the use of additional implants being used besides K-wires. Moreover, 5.4% (51/940) were lost to follow-up or excluded due to insufficient documentation, whereas 12.4% (110/889) were excluded because of anatomical regions that would have resulted in too-small subgroups for further analysis. This resulted in 779 eligible cases in the distal humerus and distal forearm (Figure 2).

The median age at the time of surgery was 8.7 years [6.0–11.8 years]. When stratified by localization, the median age for distal forearm fractures was 10.4 years [7.7–13.0 years] compared with 6.4 years [4.7–8.3 years] for distal humeral fractures (*p* < 0.001).

Overall, 480 patients (61.6%) were male and 299 (38.4%) were female, resulting in a male-to-female ratio of 1.61:1. Boys were significantly older than girls at the time of surgery, with a median age of 9.3 years [6.5–12.5 years] compared with 7.9 years [5.5–10.0 years] in girls (*p* < 0.001). Furthermore, the distal forearm fractures occurred predominantly in male patients, with 68.4% (333/487). For the distal humerus it was balanced, with 50.3% (147/292) male and 49.7% (145/292) female.

### 3.2. Fracture Patterns

With 58.5% (456/779) predominantly the left side was affected. According to the Gustilo–Anderson classification (13), 3.2% (25/779) of the fractures were categorized as I° open and 0.6% (5/779) as II° open.

Distal forearm fractures (62.5%; 487/779) were the most common and consisted of 79.9% (389/487) metaphyseal fractures (AO 23 M) and 20.1% (98/487) epiphyseal fractures (AO 23 E). Distal humeral fractures (AO 13; 37.5%; 292/779) were mainly displaced supracondylar humeral fractures 93.5% (273/292), consisting of 1.7% (5/292) type II, 24.0% (70/292) type III and 67.8% (198/292) type IV fractures according to the van Laer classification [18].

The indication for surgery was in 4.7% (37/779) due to secondary displacement following initial conservative treatment.

### 3.3. Surgical Technique

With 80.0% (623/779) most of the fractures were treated surgically on the day of accident and 13.9% (108/779) on the following day. The main reasons for the 6.1% (48/779) delayed surgeries were failed conservative treatment attempts and organizational delays (e.g., planned surgery, additional diagnostic, comorbidities). The median duration of surgery was 20.0 min [13.0–32.0 min]. This value is without the time of postoperative cast application, as this time was not specifically measured.

With 97.2% (757/779) most fractures were reduced in a closed manner, while 2.8% (22/779) needed open reduction. For the latter, wound closure was performed in 50.0% (11/22) of the cases with absorbable and in the other 50% (11/22) with non-absorbable sutures.

The K-wires were left unburied in 76.0% (592/779) while the other 24.0% (187/779) were buried below skin level. PAP was applied in 9.1% (71/779) of the cases and in 39.4% (28/71) due to an open fracture (Table 1). PAP was applied in 11.0% (32/292) of cases at the distal humerus and 8.0% (39/487) of cases at the distal forearm. With 90.1% (64/71) mainly Cefuroxime (second-generation cephalosporine) was used at the median dosage of 25.0 mg/kg [24.00–26.79 mg/kg]. Otherwise, 7.0% (5/71) received Clindamycine and 2.8% (2/71) Amoxicillin with Clavulanic acid.

### 3.4. Follow-Up and Complications

The median time from surgery to implant removal was 30 days [28–36 days]. Overall, 17.6% (137/779) of the cases had at least one documented complication, and 155 complications were documented in total. The most frequent complications were cast-related issues (5.0%; 39/779), nerve lesions/paresis (2.4%; 19/779), motor function deficits (1.7%; 13/779) and sensory disturbances (1.7%; 13/779). These complications were observed irrespective of the use of a PAP (*p* > 0.05).

Complications occurred more frequently in younger patients. The median age of patients with complications was 7.7 years [5.8–10.7 years] while the median age without complications was 8.9 years [6.1–11.9 years] (*p* = 0.029). On the one hand, infectious complications or complications of unburied K-wires showed no significant difference by age (*p* > 0.752). On the other hand, the occurrence of complications associated with buried K-wires was significantly higher in younger patients (*p* = 0.009).

The number of complications was significantly higher at the distal humerus (30.8%; 90/292) compared to the distal forearm (13.3%; 65/487) (*p* < 0.001). There was no difference in pin site infections by location (distal humerus: 1.4% (4/292); distal forearm: 1.0% (5/487); *p* = 0.735) or in wound healing disorder (distal humerus: 0.7% (2/292); distal forearm: 1.8% (9/487); *p* = 0.224). Wire perforations of buried wires (5.9%, 11/187) were observed only in distal humerus fractures. Motor function deficits (1.7%, 13/779) and nerve lesions/paresis (2.4%; 19/779) were significantly more prevalent at the distal humerus (*p* < 0.001 for both). No significant differences based on location were observed for cast problems, sensory disturbances, consolidation problems or complications associated with unburied wires (*p* > 0.05) (Table 2).

Pin site infections occurred in 3.6% (1/28) of open fracture/closed reduction cases with PAP, 3.7% (1/27) of closed fracture/closed reduction cases with PAP and 1.0% (7/700) of closed fracture/closed reduction cases without PAP. Wound healing disorders were only observed in the closed fracture/closed reduction without PAP subgroup (1.6%, 11/700). In cases of closed fractures requiring open reduction the overall complication rates were high regardless of PAP use (43.8% (7/16) with PAP and 33.3% (2/6) without PAP). Notably, none of the 22 cases treated with open reduction developed an infectious complication (Table 3). Until implant removal no case of osteomyelitis was documented.

Across subgroups defined by fracture type, reduction technique and K-wire technique, there was no significant difference in pin site infection (*p* = 0.436) and wound healing disorders (*p* = 0.755). The overall complication rate among the K-wire subgroups’ was higher when open reduction was necessary or the wires were buried (*p* = 0.013). Implant-associated complications were 10.2% (19/187) for buried and 3.4% (20/592) for unburied K-wires. Complications occurred more often when closed fractures were treated with closed reduction and buried wires (31.2%; 53/170) than unburied wires (16.3%; 91/557). Motor function deficits, sensory disturbances, and nerve lesions/paresis showed significant differences between the subgroups (*p* < 0.048). Among closed fractures treated with closed reduction, infectious complications remained uncommon with both buried and unburied wires (pin site infection: 0.6% (1/170) versus 1.3% (7/557), and wound healing disorder: 0.6% (1/170) versus 1.8% (10/557)) (Table 4).

## 4. Discussion

This retrospective study examined 779 pediatric K-wire osteosyntheses for distal forearm and distal humerus fractures. The overall complication rate was 17.6%, which is consistent with reported rates of up to 35% [14,15]. No significant association was observed between the administration of PAP and infectious complications, such as pin site infections or wound healing disorders. Furthermore, leaving K-wires unburied was associated with fewer implant-associated complications. The rates of infection, nerve lesions and motor function deficits were comparable between the two techniques.

### 4.1. Perioperative Antibiotic Prophylaxis (PAP)

Current international guidelines such as the American Society of Health-System Pharmacists (AHSP) guideline [12] and the German “Arbeitsgemeinschaft der Wissenschaftlichen Medizinischen Fachgesellschaften e. V.” (AWMF) guideline [11] recommend PAP for osteosyntheses. However, these recommendations are mainly based on expert consensus and not adapted to a pediatric population and minimally invasive surgery. A recent review by Eckmann et al. [13] showed that the decision to use PAP should be critically evaluated in clean surgical procedures with a low risk of infection, even when implants are used. Routine prophylaxis may not be beneficial in low-risk settings and should be weighed against potential side effects and the broader issue of antibiotic stewardship.

A prospective double-blinded randomized controlled trial by Gupta et al. [15] including 160 supracondylar humerus fractures treated by closed reduction and unburied K-wire pinning showed no significant difference for infectious complications if PAP or a placebo was used.

Four retrospective studies and one randomized controlled trial covering both data of pediatric and adult population in different anatomic locations were included in a review that showed no significant advantage of PAP for unburied K-wires [19]. However, whether wires were buried or left unburied was not the focus of the review.

Bhatt et al. [20] performed a retrospective analysis of pediatric supracondylar humerus fractures treated with K-wires, where a majority of 83.4% (755/905) received PAP. The results were similar to those of this study, with an infection rate of 2.5% without PAP and 2.0% with PAP, showing no significant difference. Bloomer et al. [21] had a more balanced ratio, with 44.6% (337/845) of patients receiving PAP for the same fracture and treatment type. However, the reported infection complications were lower: 0.4% with PAP and 0.6% without PAP.

A multicenter cohort study that included 9054 orthopedic procedures also questioned the effectiveness of PAP in pediatric surgery in reducing SSIs, as it showed no correlation between PAP and SSI rates [22].

Taken together, neither the literature nor the data from this study support the general use of PAP for closed fractures in children treated with closed reduction and K-wire osteosyntheses.

### 4.2. Surgical Technique

Burying of K-wires is often carried out with the intention of reducing the risk of implant-associated infections, which was shown in a survey among German pediatric and trauma surgeons [23]. However, a review of 15 studies involving 1854 patients revealed a comparable low infection rate of 1.74% for buried and 2.07% for unburied K-wires [24]. This is consistent with the results of this study. A meta-analysis of hand fractures demonstrated a lower risk of infection with buried K-wires, although the included studies were conducted predominantly in adult populations with different healing potential [25]. Lateral condyle fractures have reported K-wire perforation rates of 16.0% to 23.0% [24,26]. In our cohort, perforation of buried K-wires was observed only in 5.9% of the cases. However, comparability is limited as only supracondylar fractures were assessed in this study. Lateral condyle fractures are treated with screw osteosyntheses at our institution. Nevertheless, implant-associated complications were higher for unburied wires in this analysis.

The main benefit of unburied K-wires is that they can be removed in an outpatient clinic, sparing children a second general anesthetic and reducing the cost of implant removal [4,23,24,25,26,27]. Counterintuitively, the removal of unburied wires was not associated with higher distress and well accepted by parents in a comparative prospective study including 291 patients [9].

Considering the results and the given literature, the unburied K-wire technique has a similar overall complication rate and even fewer implant-associated complications. Furthermore, the removal of the K-wires without general anesthesia is a great advantage.

### 4.3. Limitations

This study has several limitations. Its retrospective, single-center design may limit generalizability. No standardized surgical or follow-up protocol was applied. Although documentation quality was generally high, a loss of information due to insufficient documentation cannot be fully ruled out. Procedures were performed by multiple surgeons, including the authors. However, systematic bias in documentation or outcome assessment is considered unlikely. Decisions regarding perioperative antibiotic prophylaxis (PAP) and whether K-wires were buried or unburied generally followed institutional standards, although individual deviations occurred. In addition, while the institutional standard changed from buried to unburied K-wires in 2017, adherence to this change was not uniform throughout the study period. Despite exclusion of rare anatomical regions, subgroup sizes remained heterogeneous, limiting comparability. Because PAP- and K-wire-related complications were rare, statistical power was limited and statistical tests like multivariable logistic regression to further evaluate cofounders could not be used. Follow-up was restricted to implant removal, and although unlikely, late complications cannot be completely excluded.

## 5. Conclusions

The omission of perioperative antibiotic prophylaxis in closed reduction and K-wire osteosyntheses in pediatric and adolescent patients does not alter the risk for infectious complications. Moreover, leaving K-wires unburied seems to have fewer implant-associated complications. Furthermore, it allows implant removal in an outpatient setting without the need for a second general anesthetic.

## Figures and Tables

**Table 1:** Descriptive characteristics of fracture type, reduction technique, perioperative antibiotic prophylaxis (PAP) and K-wire technique in the study population. All numbers are shown as n (%) and refer to the total study population (n = 779).

Fracture Type	Reduction Technique	PAP		K-Wires	
		Not Applied	Applied	Buried	Unburied
779 (100)	779 (100.0)	708 (90.9)	71 (9.1)	187 (24.0)	592 (76.0)
Closed fracture	Closed				
749 (96.1)	727 (93.3)	700 (89.9)	27 (3.5)	170 (21.8)	557 (71.5)
	Open				
	22 (2.8)	6 (0.8)	16 (2.1)	10 (1.3)	12 (1.5)
Open fracture	Closed				
30 (3.9)	30 (3.9)	2 (0.3)	28 (3.6)	7 (0.9)	23 (3.0)
	Open				
	0	0	0	0	0

PAP = perioperative antibiotic prophylaxis; buried K-wire = left beneath skin level; unburied K-wire = left percutaneously protruding.

**Table 2:** Complications stratified by fracture localization in the study population (n = 779).

Region	Distal Humerus	Distal Forearm		
AO region	13	23	Total n (%)	*p*-value
Number n (%)	**292 (37.5)**	**487 (62.5)**	**779 (100)**	
PAP associated complications				
Pin site infection	4 (1.4)	5 (1.0)	**9 (1.2)**	0.735
Wound healing disorder	2 (0.7)	8 (1.6)	**10 (1.3)**	0.335
Buried K-wire associated complications (n = 187)				
K-wire perforation	11 (3.8)	0	**11 (1.4)**	0.001
Irritation	2 (0.7)	1 (0.2)	**3 (0.4)**	0.576
Implant failure	4 (1.4)	1 (0.2)	**5 (0.6)**	0.254
Unburied K-wire associated complications (n = 592)				
Irritation	0	3 (0.6)	**3 (0.4)**	0.555
Ingrowth below skin level	2 (0.7)	7 (1.4)	**9 (1.2)**	0.726
Implant failure	4 (1.4)	4 (0.8)	**8 (1.0)**	0.274
Other complications				
Motor function deficit	12 (4.1)	1 (0.2)	**13 (1.7)**	<0.001
Sensory disturbance	8 (2.7)	5 (1.0)	**13 (1.7)**	0.086
Nerve lesions/paresis	19 (6.5)	0	**19 (2.4)**	<0.001
Consolidation insufficient	0	6 (1.2)	**6 (0.8)**	0.089
Consolidation in malposition	4 (1.4)	2 (0.4)	**6 (0.8)**	0.204
Cast problems	18 (6.2)	21 (4.3)	**39 (5.0)**	0.308
Sum of complications	90 (30.8)	66 (13.6)	**156 (19.5)**	<0.001

Values are presented as n (%). Percentages refer to each subgroup. AO regions shown: 13 (distal humerus), 23 (distal forearm). *p*-Values were calculated using Pearson’s Chi-square test or Fisher’s exact test, as appropriate (two-sided). For the variable “Sum of complications” the Mann–Whitney U test was applied. Statistical significance was defined as *p* < 0.05. Significant *p*-values are highlighted in red.

**Table 3:** Complications stratified by fracture type, reduction technique and perioperative antibiotic prophylaxis (PAP) in the study population (n = 779).

Group	Open Fracture *Closed Reduction* PAP Applied	Open Fracture *Closed Reduction* PAP Not Applied	Closed Fracture *Open Reduction* PAP Applied	Closed Fracture *Open Reduction* PAP Not Applied	Closed Fracture *Closed Reduction* PAP Applied	Closed Fracture *Closed Reduction* PAP Not Applied	Total n (%)	*p*-Value
Number n (%)	**28 (3.6)**	**2 (0.3)**	**16 (2.1)**	**6 (0.8)**	**27 (3.5)**	**700 (90.9)**	**779 (100)**	
PAP associated complications								
Pin site infection	1 (3.6)	0	0	0	1 (3.7)	7 (1.0)	**9 (1.2)**	0.269
Wound healing disorder	0	0	0	0	0	10 (1.4)	**10 (1.3)**	1.000
Buried K-wire associated complications (n = 187)								
K-wire perforation	1 (3.6)	0	0	0	0	10 (1.4)	**11 (1.4)**	0.758
Irritation	0	0	0	0	0	5 (0.7)	**3 (0.4)**	1.000
Implant failure	0	0	0	0	0	3 (0.4)	**5 (0.6)**	1.000
Unburied K-wire associated complications (n = 592)								
Irritation	0	0	0	0	0	3 (0.4)	**3 (0.4)**	1.000
Ingrowth below skin level	0	0	0	0	1 (3.7)	8 (1.1)	**9 (1.2)**	0.601
Implant failure	0	0	1 (6.3)	0	0	7 (1.0)	**8 (1.0)**	0.262
Other complications								
Motor function deficit	0	0	2 (12.5)	0	0	11 (1.6)	**13 (1.7)**	0.136
Sensory disturbance	0	0	1 (6.3)	1 (16.7)	0	11 (1.6)	**13 (1.7)**	0.109
Nerve lesions/paresis	0	0	1 (6.3)	1 (16.7)	0	17 (2.4)	**19 (2.4)**	0.221
Consolidation insufficient	0	0	0	0	0	6 (0.9)	**6 (0.8)**	1.000
Consolidation in malposition	0	0	1 (6.3)	0	0	5 (0.7)	**6 (0.8)**	0.225
Cast problems	1 (3.6)	0	1 (6.3)	0	2 (7.4)	35 (4.7)	**39 (5.0)**	0.835
Sum of all complications	3 (10.7)	0	7 (43.8)	2 (33.3)	4 (14.8)	140 (20.0)	**156 (19.5)**	0.350

Values are presented as n (%). Percentages refer to each subgroup. *p*-Values were calculated using Chi-square test or Fisher’s exact test with Monte Carlo simulation, as appropriate (two-sided). Statistical significance was defined as *p* < 0.05.

**Table 4:** Complications stratified by fracture type, reduction technique and K-wire technique (buried vs. unburied) in the study population (n = 779).

Group	Open Fracture *Closed Reduction* K-Wire Buried	Open Fracture *Closed Reduction* K-Wire Unburied	Closed Fracture *Open Reduction* K-Wire Buried	Closed Fracture *Open Reduction* K-Wire Unburied	Closed Fracture *Closed Reduction* K-Wire Buried	Closed Fracture *Closed Reduction* K-Wire Unburied	Total n (%)	*p*-Value
Number n (%)	**7 (0.9)**	**23 (3.0)**	**10 (1.3)**	**12 (1.5)**	**170 (21.8)**	**557 (71.5)**	**779 (100)**	
PAP associated complications								
Pin site infection	0	1 (4.3)	0	0	1 (0.6)	7 (1.2)	**9 (1.2)**	0.436
Wound healing disorder	0	0	0	0	0	10 (1.8)	**10 (1.3)**	0.441
Buried K-wire associated complications (n = 187)								
K-wire perforation	1 (14.2)		0		10 (5.9)		**11 (1.4)**	0.426
Irritation	0		0		5 (2.9)		**3 (0.4)**	1.000
Implant failure	0		0		3 (1.8)		**5 (0.6)**	1.000
Unburied K-wire associated complications (n = 592)								
Irritation		0		0		3 (0.5)	**3 (0.4)**	1.000
Ingrowth below skin level		0		0		9 (1.6)	**9 (1.2)**	1.000
Implant failure		0		1 (8.3)		7 (1.3)	**8 (1.0)**	0.183
Other complications								
Motor function deficit	0	0	2 (20.0)	0	5 (2.9)	6 (1.1)	**13 (1.7)**	0.022
Sensory disturbance	0	0	0	2 (16.7)	5 (2.9)	6 (1.1)	**13 (1.7)**	0.030
Nerve lesions/paresis	0	0	1 (10.0)	1 (8.3)	8 (4.7)	9 (1.6)	**19 (2.4)**	0.048
Consolidation insufficient	0	0	0	0	0	6 (1.1)	**6 (0.8)**	0.568
Consolidation in malposition	0	0	0	1 (8.3)	0	5 (0.9)	**6 (0.8)**	0.196
Cast problems	0	1 (4.3)	0	1 (8.3)	15 (8.8)	22 (3.9)	**39 (5.0)**	0.164
Sum of all complications	1 (14.2)	2 (8.7)	3 (30.0)	6 (50.0)	53 (31.2)	91 (13.0)	**156 (19.5)**	0.013

Values are presented as n (%). Percentages refer to each subgroup. *p*-Values were calculated using Chi-square test or Fisher’s exact test with Monte Carlo simulation, as appropriate (two-sided). Statistical significance was defined as *p* < 0.05. Significant *p*-values are highlighted in red.

## Data Availability

The original contributions presented in this study are included in the article. Further inquiries can be directed to the corresponding author.

## References

[1] Huber W. (2008). Historical remarks on Martin Kirschner and the development of the Kirschner (K)-wire. Indian J. Plast. Surg..

[2] Slongo T. (2020). Technik und Biomechanik der Bohr-Draht(Kirschner-Draht)-Osteosynthese bei Kindern. Oper. Orthop. Traumatol..

[3] Subramanian P., Kantharuban S., Shilston S., Pearce O.J. (2012). Complications of Kirschner-Wire Fixation in Distal Radius Fractures. Tech. Hand Up. Extrem. Surg..

[4] Ormsby N.M., Walton R.D.M., Robinson S., Brookes-Fazakerly S., Yuen Chang F., McGonagle L. (2016). Buried versus unburied Kirschner wires in the management of paediatric lateral condyle elbow fractures: A comparative study from a tertiary centre. J. Pediatr. Orthop. B.

[5] Qin Y.F., Li Z.J., Li C.K., Bai S.C., Li H. (2017). Unburied versus buried wires for fixation of lateral condyle distal humeral fractures: A meta-analysis. Medicine.

[6] Koç T., Ahmed J., Aleksyeyenko S. (2012). Buried Kirschner wires in hand trauma: Do they reduce infection rates and is it worth the extra cost?. Eur. J. Plast. Surg..

[7] Khaled M., Fadle A.A., Hassan A.A.M., Khalifa A.A.M., Nabil A.M., Hafez A., Oyoun N.A. (2023). To Bury or Not to Bury the K-Wires After Fixation of Both Bone Forearm Fractures in Patients Younger Than 11 Years Old: A Randomized Controlled Trial. J. Pediatr. Orthop..

[8] Suganuma S., Tada K., Takagawa S., Yasutake H., Shimanuki K., Shinmura K., Fujita K., Tsuchiya H. (2022). Comparing exposed and buried Kirschner wires in fixation for pediatric supracondylar humerus fractures: A propensity score-matched study. J. Orthop. Surg..

[9] Schneidmueller D., Eijkenboom A., Brand A., Langenhan R., Kertai M., Voth M. (2022). To bury or not to bury—Kirschner wire fixation in children and adolescents. Dtsch. Ärztebl. Int..

[10] Nthumba P.M., Huang Y., Perdikis G., Kranzer K. (2022). Surgical Antibiotic Prophylaxis in Children Undergoing Surgery: A Systematic Review and Meta-Analysis. Surg. Infect..

[11] Deutsche Gesellschaft für Hygiene und Mikrobiologie e.V. (2024). S3-Leitlinie Perioperative und Periinterventionelle Antibiotikaprophylaxe—Aktualisierung 2024. Langversion. Version 5.0. AWMF-Registernummer: 067-009 [Internet]. https://register.awmf.org/de/leitlinien/detail/067-009.

[12] Bratzler D.W., Dellinger E.P., Olsen K.M., Perl T.M., Auwaerter P.G., Bolon M.K. (2013). Clinical Practice Guidelines for Antimicrobial Prophylaxis in Surgery. Surg. Infect..

[13] Eckmann C., Aghdassi S.J.S., Brinkmann A., Pletz M., Rademacher J. (2024). Perioperative antibiotic prophylaxis—Indications and modalities for the prevention of postoperative wound infection. Dtsch. Ärztebl. Int..

[14] Leeuwen W.F., van Hoorn B.T.J.A., Chen N., Ring D. (2016). Kirschner wire pin site infection in hand and wrist fractures: Incidence rate and risk factors. J. Hand Surg. Eur. Vol..

[15] Gupta S.K., Esposito E.R., Phillips R., Schwab P.E., Leary E.V., Hoernschemeyer D.G. (2024). Effect of Antibiotic Prophylaxis on Infection Rates in Supracondylar Humerus Fractures Treated with Closed Reduction and Percutaneous Pinning: A Prospective Double-Blinded Randomized Controlled Trial. J. Am. Acad. Orthop. Surg..

[16] Slongo T.F., Audigé L., AO Classification Group (2007). Fracture and dislocation classification compendium for children: The AO comprehensive classification of long bone fractures (PCCF). J. Orthop. Trauma.

[17] Gustilo R., Anderson J. (1976). Prevention of infection in the treatment of one thousand and twenty-five open fractures of long bones: Retrospective and prospective analyses. J. Bone Jt. Surg..

[18] Lutz N., Audigé L., Schmittenbecher P., Clavert J.M., Frick S., Slongo T. (2011). Diagnostic Algorithm for a Validated Displacement Grading of Pediatric Supracondylar Fractures. J. Pediatr. Orthop..

[19] Abul A., Karam M., Al-Shammari S., Giannoudis P., Pandit H., Nisar S. (2023). Peri-operative Antibiotic Prophylaxis in K-Wire Fixation: A Systematic Review and Meta-Analysis. Indian J. Orthop..

[20] Bhatt E., Ridley T.J., Kruckeberg B., Quanbeck Z., Quanbeck D.S., Schiffern A. (2021). Efficacy of Antibiotics in Supracondylar Fractures. J. Pediatr. Orthop..

[21] Bloomer A.K., Coe K.M., Brandt A.M., Roomian T., Brighton B., Scannell B.P. (2022). Hold the Antibiotics: Are Preoperative Antibiotics Unnecessary in the Treatment of Pediatric Supracondylar Humerus Fractures?. J. Pediatr. Orthop..

[22] He K., Nayak R.B., Allori A.C., Brighton B.K., Cina R.A., Ellison J.S., Goretsky M.J., Jatana K.R., Proctor M.R., Grant C. (2022). Correlation Between Postoperative Antimicrobial Prophylaxis Use and Surgical Site Infection in Children Undergoing Nonemergent Surgery. JAMA Surg..

[23] Schneidmueller D., Kertai M., Bühren V., Von Rüden C. (2018). Kirschner-Draht-Osteosynthese bei Frakturen im Kindesalter: Drähte versenken oder nicht?: Ergebnisse einer Umfrage zur Versorgungsrealität in Deutschland. Unfallchirurg.

[24] Pullan J., Ayeko O., Metcalfe J. (2025). Buried or exposed kirschner wires in paediatric upper extremity fracture fixation: A systematic review and meta-analysis of infection rates and complications. Injury.

[25] Sahib Y., Alsadoun L. (2026). Buried Versus Exposed K-Wires in Hand Fracture Fixation: A Meta-Analysis of Outcomes. Cureus.

[26] Das De S., Bae D.S., Waters P.M. (2012). Displaced Humeral Lateral Condyle Fractures in Children: Should We Bury the Pins?. J. Pediatr. Orthop..

[27] Eckhoff M.D., Tadlock J.C., Nicholson T.C., Wells M.E., Garcia E., Hennessey T.A. (2022). VanOpen reduction of lateral condyle fractures: A systematic review. Shoulder Elb..

